# Drug Screening of Primary Human Endometriotic Cells Based on Micro‐Encapsulating Microfluidic Chip

**DOI:** 10.1002/advs.202504647

**Published:** 2025-04-28

**Authors:** Qiong Chen, Jing Wang, Wenzhao Li, Luoran Shang, Dexuan Wang, Ping Duan

**Affiliations:** ^1^ Department of Obstetrics and Gynecology The Second Affiliated Hospital and Yuying Children's Hospital of Wenzhou Medical University Wenzhou 325027 China; ^2^ Oncology Discipline Group The Second Affiliated Hospital and Yuying Children's Hospital of Wenzhou Medical University Wenzhou 325027 China; ^3^ Shanghai Xuhui Central Hospital Zhongshan‐Xuhui Hospital and the Shanghai Key Laboratory of Medical Epigenetics International Co‐laboratory of Medical Epigenetics and Metabolism (Ministry of Science and Technology Institutes of Biomedical Sciences) Fudan University Shanghai 200032 China; ^4^ Wenzhou Institute University of Chinese Academy of Sciences Wenzhou 325001 China; ^5^ Department of Biomedical Engineering The Hong Kong Polytechnic University Hong Kong SAR 999077 China; ^6^ Department of Pediatrics The Second Affiliated Hospital and Yuying Children's Hospital of Wenzhou Medical University Wenzhou 325027 China

**Keywords:** drug screening, endometriosis, hydrogel, microcapsule, microfluidics

## Abstract

Endometriosis (EMs), a significant global health issue, characterized by unclear pathogenesis, nonspecific symptoms, and poor treatment outcomes. The organ‐on‐chip technology has achieved great advances in disease modeling, yet its potential in EMs‐related research remains largely untapped. Herein, a microfluidic chip platform that integrates primary cell‐laden microcapsules for personalized drug evaluation. Specifically, primary human ectopic endometrial stromal cells (hESCs) within microcapsules featuring a biocompatible carboxymethyl cellulose (CMC) core and a stable alginate (ALG) shell using precise microfluidic electrospray are encapsulated. These microcapsules are integrated into a chip with a branched gradient generator and multiple cell‐culture chambers, enabling tailored and high‐throughput drug screening. By exposing hESCs‐microcapsules derived from primary cells of distinct patient individuals to various drugs on‐chip, significant inter‐individual variability was revealed, with a strong correlation to clinical outcomes. This unique combination of patient‐specific 3D microenvironments and dynamic drug gradient control represents a paradigm shift in personalized EMs research. Further integrating with omics techniques, its capability in exploring promising drugs is showcased. These results reveal that the chip platform could deliver dependable and personalized drug screening outcomes, thereby benefiting both scientific inquiries and clinical therapies.

## Graphical abstract

To address the challenges of unclear mechanisms and poor therapeutic efficacy in endometriosis (EMs), this study develops a microfluidic chip platform integrated with primary cell‐laden microcapsules. Gradient drug screening reveals individualized treatment response variations among patients and their correlation with clinical outcomes. Combined with omics technologies, this platform offers a reliable tool for personalized drug evaluation, advancing precision medicine.

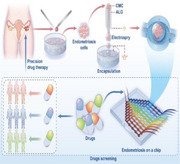

## 1 Introduction

Endometriosis (EMs) stands as a significant global health issue, with an estimated 190 million women worldwide enduring the associated symptoms including dysmenorrhea, difficulties with defecation and urination, and even infertility.^[^ ^1^, ^2^, ^3^ ^]^ The prevailing treatment of EMs involves surgery and maintenance therapy with hormonal medications, but the outcomes are often unsatisfactory, with high recurrence rate and the requirement of additional therapy.^[^ ^4^, ^5^, ^6^, ^7^, ^8^ ^]^ Basically, the unclear mechanisms, nonspecific symptoms, and poor treatment outcomes of EMs present significant barriers, creating an urgent need for effective disease modeling and drug evaluation. Currently, various models have been explored, including cell culture models, animal models, organ‐on‐a‐chip systems, etc. Conventional cell‐based models cannot sufficiently recapitulate critical physiological parameters including the biophysical and biochemical microenvironment, 3D tissue‐specific architecture, and vascular perfusion dynamics, thus limiting their capacity to faithfully replicate human pathophysiology.^[^ ^9^ ^]^ Meanwhile, preclinical animal models are constrained by intrinsic interspecies variability and ethical concerns, and exhibit limited fidelity in modeling complex human disease mechanisms.^[^ ^10^ ^]^ Among these approaches, the organ‐on‐a‐chip technique has received increasing attention due to its ability in simulating human physiology and bypassing the ethical concerns. Although with exciting progress, existing organ‐on‐a‐chip drug screening platforms predominantly focus on major organs of drug metabolism/toxicity, while their applications in EMs‐related research remain limited.^[^ ^10^, ^11^, ^12^, ^13^ ^]^ Therefore, there is still an urgent need for a specialized and effective EMs drug evaluation model.

**Figure 1:**
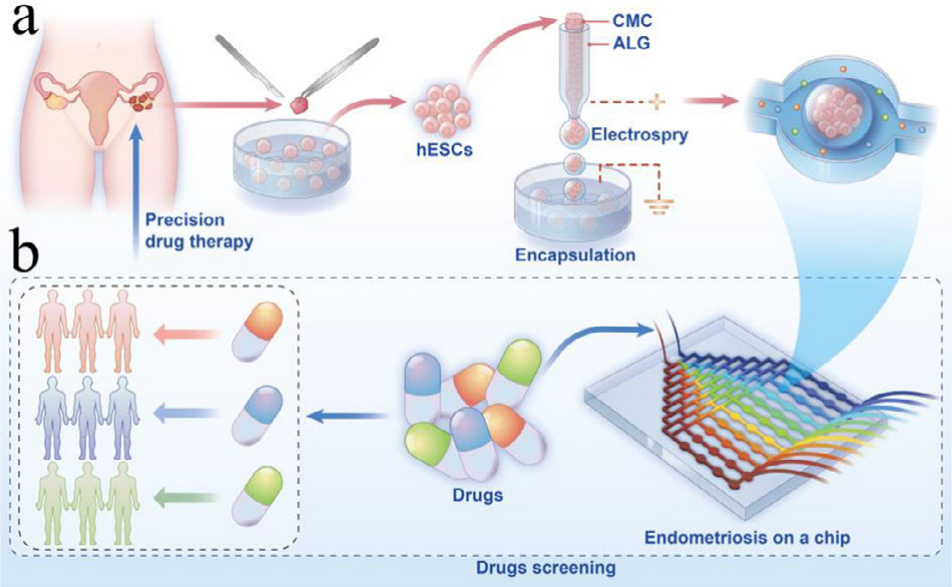
Schematic diagram of drug evaluation in the EMs‐on‐a‐chip platform. a) Synthesis of hESCs‐encapsulated microcapsules. Primary endometriosis stromal cells derived from patients were mixed with a CMC solution and encapsulated into cell‐laden microcapsules with a CMC core and an alginate shell using microfluidic electrospray technology. b) The microfluidic chip integrated with hESCs‐encapsulated microcapsules for drug screening.

Herein, we introduce a microfluidic chip system that integrates microcapsules containing primary human ectopic endometrial stromal cells (hESCs) for personalized assessment of drug efficacy, as illustrated in **Figure** **1**. Organ‐on‐a‐chip technology provides a microenvironment that closely mimics in vivo conditions, surpassing traditional 2D cell cultures and offering distinct advantages for the cultivation of human tissues. The precision engineering methods enable faithful reconstruction of human‐specific biological complexity, including dynamic cell‐cell communication networks, functional tissue‐tissue interfaces, and spatiotemporal control over biophysical microenvironments.^[^ ^14^, ^15^, ^16^, ^17^ ^]^ Furthermore, through intricate design of the microfluidic channel geometry, specific physicochemical environments such as drug concentration gradients and biomimetic microstructures can be created, which is conducive to studying customized interactions.^[^ ^18^, ^19^, ^20^, ^21^, ^22^, ^23^ ^]^ However, successfully replicating hESCs growth environment on microfluidic chips continues to pose a significant challenge. In this context, microcapsules can serve as highly adaptable and robust microcarriers for the encapsulation of cells and cell spheroids with preservation of high cellular viability. Notably, the size, shape, and material component of the microcapsules can be tailored to facilitate substance transfer between the external environment and cells encapsulated within. Consequently, the development of a microfluidic chip integrated with hESCs‐laden microcapsules could offer a promising strategy for creating an effective in vitro model tailored for EMs drug evaluation.

To actualize this vision, we employed the microfluidic electrospray technology to load primary hESCs within hydrogel microcapsules. These cell‐laden microcapsules were subsequently integrated into a microfluidic chip for spheroid formation and drug screening. The precision of electrospray technology ensured the uniformity in structure and size of the microcapsules. Carboxymethyl cellulose (CMC) served as the biocompatible core, while alginate (ALG) constituted the stable shell of the capsules, effectively and safely encapsulating and supporting the cells. The microfluidic chips, featuring a branched gradient drug concentration generator and multiple chambers for accommodating cell spheroid microcapsules, facilitated customized and high‐throughput drug screening processes. In practical demonstrations, we exposed spheroids derived from primary cells of distinct clinical individuals to various drugs on the chip. The results revealed significant inter‐individual variability in response to the same treatment, highlighting the complexities of patient‐specific drug sensitivity. The data also demonstrated a strong correlation between chip results and clinical outcomes, affirming the precision and relevance. By further integrating this chip platform with advanced techniques such as omics, we showcased its capability in exploring promising drugs. All these features underscored the value of such a chip platform in providing reliable and accurate drug evaluation results, serving both scientific research and clinical treatment.

## 2 Results and Discussion

**Figure 2:**
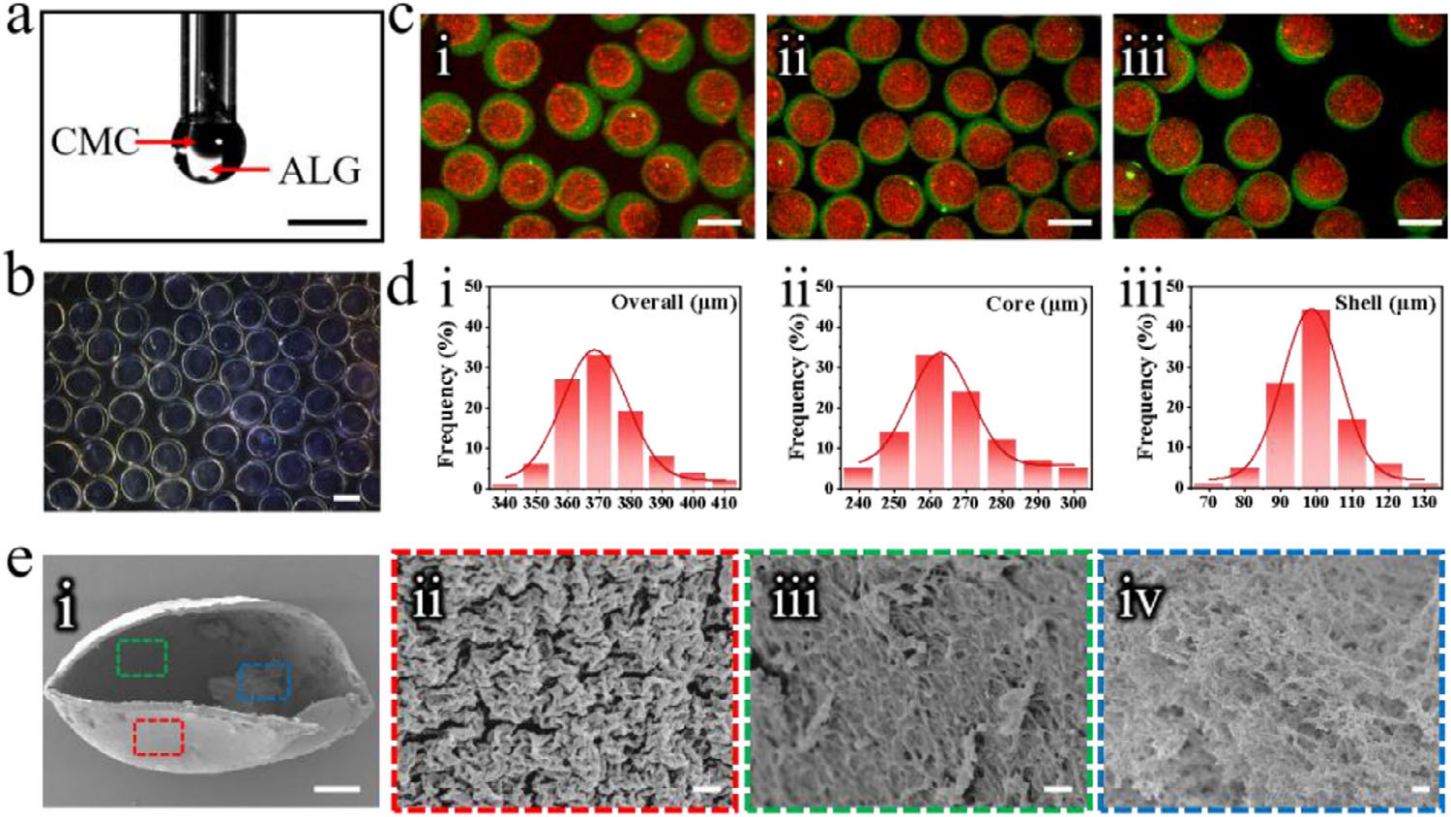
Fabrication and characterization of CMC/ALG microcapsules. a) Imaging of the CMC/ALG droplet creation process. b) Microcapsules observed through an optical microscope. c) Microcapsules with ALG mixed with green fluorescent nanoparticles, and CMC mixed with red fluorescent nanoparticles. From left to right, the microcapsules were fabricated under varying inner and outer phase flow rates (µL min^−1^). i (*F*_inner =_ 4, *F*_outer =_ 20), ii (*F*_inner =_ 8, *F*_outer =_ 20), iii (*F*_inner =_ 10, *F*_outer =_ 20). d) The particle size distribution of CMC/ALG microcapsules, when the outer phase flow rate was 20 µL min^−1^, the inner phase flow rate was 4 µL min^−1^, the voltage was 7 kV, and the collection liquid height was 5 cm. i (Overall), ii (Core), iii (Shell). *n* = 100. e) SEM images of a microcapsule: i) a cleaved microcapsule after supercritical treatment, ii) the outer layer of the microcapsule, iii) the internal surface of the microcapsule, and iv) CMC core. Scale bars are 0.5 µm in (e, ii, g, iii), 1 µm in (e, iv), 50 µm in (e, i), 300 µm in (b, c), and 500 µm in (a).

We initially fabricated cell‐laden hydrogel microcapsules. The microcapsules featured a crosslinked ALG shell and a CMC core. This design facilitated the maintenance of internal stability through the ALG shell while allowing cell loading within the CMC core. The aforementioned microcapsules were derived using the microfluidic electrospray technology. To achieve such core‐shell structure, we constructed a coaxial microfluidic device. CMC and ALG solutions flowed through the inner and outer phase channels, respectively, with calcium chloride (CaCl_2_) serving as the collection medium. Under the action of an electric field, stable core‐shell microdroplets with an ALG shell and a CMC core were generated (**Figure** **2**a). After collection, the ALG shell of the droplets were rapidly crosslinked with CaCl_2_, yielding stable and uniformly sized CMC/ALG microcapsules (Figure 2b). This efficient ionic crosslinking process ensured the secure encapsulation of the internal CMC, thereby creating a sealed and stable environment for subsequent cell culture.

By manipulating the parameters of the inner and outer phase fluids, we were able to adjust the scale characteristics of the resulting microcapsules. Within the experimental range, when the internal phase flow rate remained unchanged, boosting the external phase flow rate results in an increased overall diameter of the microcapsule, while simultaneously causing a reduction in the core diameter (Figure S1a, Supporting Information). Conversely, when the flow rate of external remained constant, boosting the flow rate of internal phase resulted in a corresponding enlargement of both the overall diameter and the core diameter of the microcapsule (Figure S1b, Supporting Information). We labeled the shell with green and the core with red fluorescent nanoparticles, visually demonstrating that varying the inner and outer flow rates could adjust the core and shell diameters of the microcapsules (Figure 2c). Additionally, the dimensions of the microcapsules were also influenced by the position of the collection liquid and the applied voltage (Figure S1c,d, Supporting Information).

After determining all the aforementioned parameters, the overall diameter of the microcapsules was concentrated between 360 µm and 380 µm, the core diameter was concentrated between 250 µm and 280 µm, and the shell thickness was concentrated between 90 µm and 110 µm (Figure 2d). These results fully illustrated the precise controllability and uniformity of the microcapsules sizes. Subsequently, we characterized the core‐shell structure of the microcapsules using scanning electron microscopy (SEM) (Figure 2e). The microcapsules possessed an intact shell and exhibited enduring stability; even after five days soaking in the culture medium, the structure and shape of the microcapsules remained largely unchanged, thereby providing a stable environment for subsequent cell culture (Figure S2, Supporting Information).

**Figure 3:**
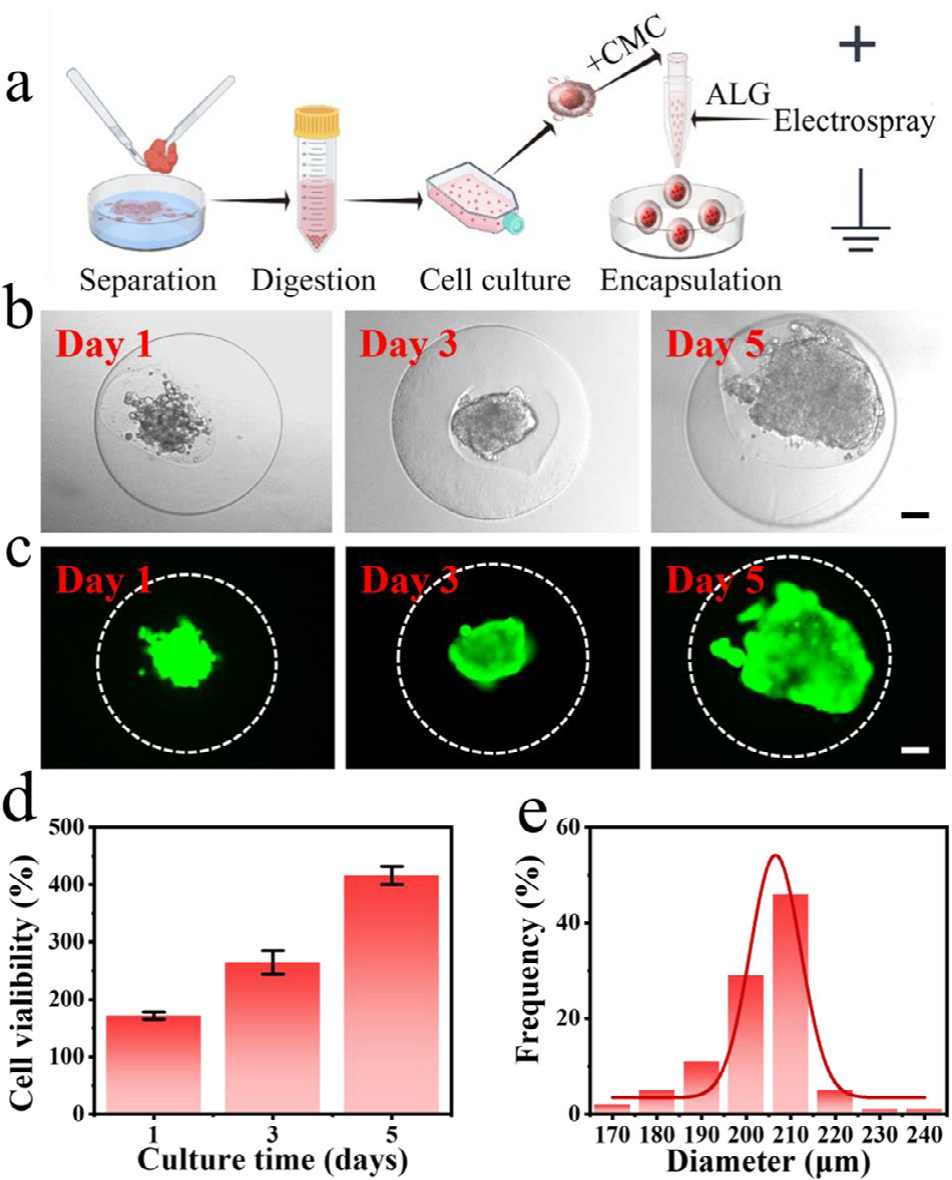
Development and analysis of hydrogel microcapsules containing EMs spheroids. a) Illustration of the isolation of primary hESCs and the creation of cell‐encapsulated microcapsules using electrospray technology (by figdraw.com). b) Bright‐field microscopic images of hESCs‐encapsulated microcapsules captured on days 1, 3, and 5 of the culture period. c) Live‐dead staining of hESCs encapsulated microcapsules performed on days 1, 3, 5. d) Quantitative proliferation analysis of EMs cells within microcapsules utilizing the CCK‐8 assay. (*n* = 10 in each group). e) On day 5 of cultivation, the size distribution of hESCs spheroids. (*n* = 100 per group). Scale bars: 50 µm in (b, c). Mean ± SD is used to present the data.

Subsequently, we utilized microcapsules as a 3D scaffold for the cultivation of primary hESCs. To acquire the primary cells, we initially collected ovarian endometriotic cyst tissues from surgically resected specimens, minced the tissue into small fragments, and digested it with Type IV collagenase (Figure S3a, Supporting Information). Subsequently, the harvested cells were identified through immunofluorescence using the specific marker Vimentin, and the results confirmed that the obtained cells were hESCs (Figure S3b, Supporting Information). Thereafter, we combined the CMC solution with the cells as the inner phase and prepared cell‐loaded microcapsules via electrospray (**Figure** **3**a). The cell‐laden microcapsules were cultured in a medium for five days. On the second day, cells spontaneously aggregated into clusters within the microcapsules core. As time progressed, these clusters grew as the cells proliferated, eventually forming spherical masses (Figure 3b). During the cultivation process, a live‐dead cell staining assay was conducted on the cells within the microcapsules, the majority of viable cells exhibited green fluorescence, whereas a small number of dead cells appeared red. The results demonstrated that the cells within the microcapsules possessed robust proliferative activity (Figure 3c). Quantitative cell proliferation experiments further confirmed this observation (Figure 3d). On the fifth day of cell microcapsule cultivation, we measured the dimensions of the cell spheroids, confirming their relatively uniform size (Figure 3e). The aforementioned results confirmed that the hydrogel microcapsules exhibited excellent biocompatibility and the capacity to support the formation of cellular spheroids, which meet the requirements for EMs‐on‐a‐chip construction.

**Figure 4:**
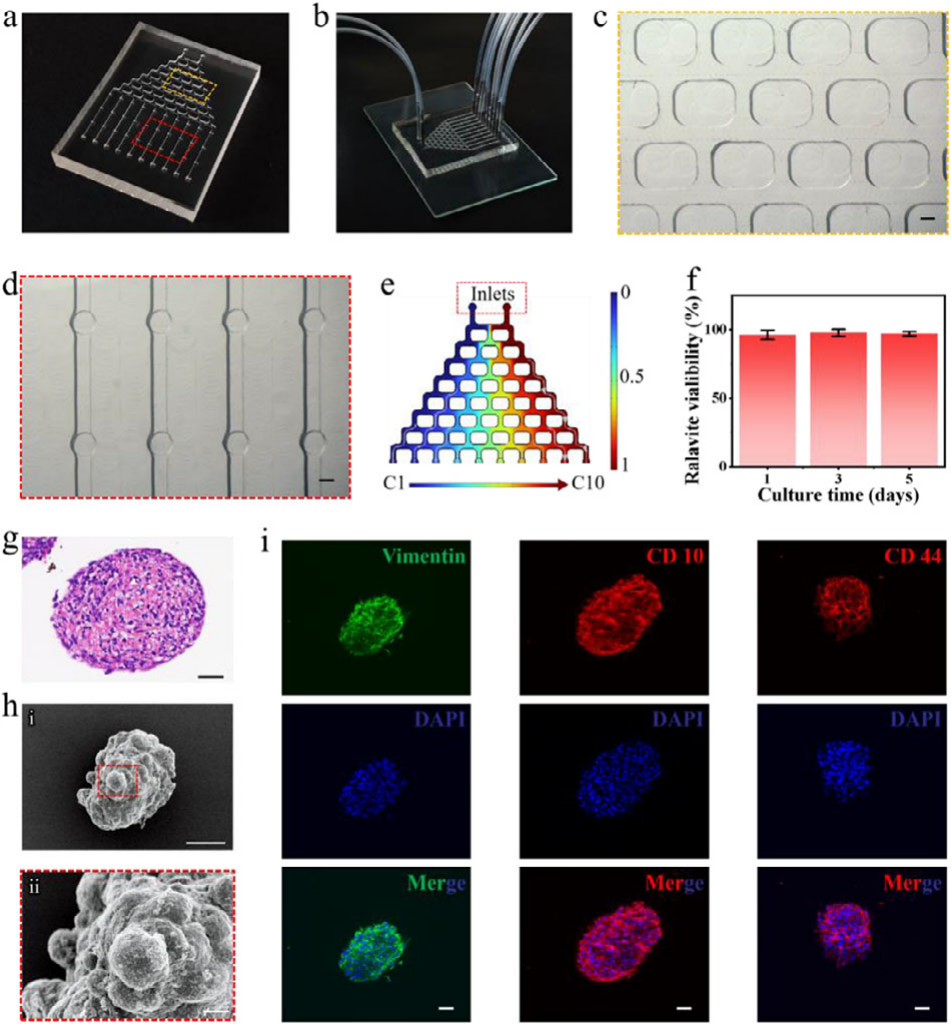
The structure of the microfluidic chip and its integration with cell spheroids‐laden microcapsules. a) A photograph of the microfluidic chip. b) A photograph of the microfluidic chip showing the inlets and outlets for medium flow. The dual tubes situated on the left serve as inflow conduits, while the ten tubes on the right function as outflow passages. c) Optical micrograph of a concentration gradient generator device. d) Microscopic image depicting the cell culture chambers. e) Numerical modeling of drug concentration distribution in a microfluidic gradient generator. f) Quantitative viability assessments of cells cultivated on the chip at 1, 3, and 5 days. (*n* = 30 in each group). g) H&E staining images of hESCs spheroids. h) SEM images of hESCs spheroids. i) Immunofluorescence staining of hESCs spheroids for specific markers: Vimentin, CD10, and CD44. Scale bars represent 10 µm in (h, ii), 50 µm in (g, h, i, i), and 500 µm in (c, d).

Concurrently, we developed a microfluidic chip platform to culture the aforementioned microcapsules and conduct subsequent drug response assays. The chip features two primary structures: a branched concentration gradient generator and ten columns of independent cell reaction chambers for accommodating the cell spheroids‐laden microcapsules (**Figure** **4**a). In operation, culture medium or medium containing drugs is introduced separately through two inlets, passes through the concentration gradient generator, proceeds to the reaction chambers, and finally exits the chip via ten outlets (Figure 4b). Specifically, the upstream concentration gradient generator branches from an initial two channels to a total of ten channels (C1 to C10), which could produce ten distinct gradient drug concentrations (Figure 4c). Downstream of the chip, the cell reaction chamber is strategically positioned at the termini of the concentration gradient generator's branches. Each branch terminus was connected to three cell culture chambers aligning in a column to accommodate microcapsules, facilitating interactions with drugs of varying concentrations (Figure 4d). To validate this, we first performed numerical simulations of drug concentrations within the chip, revealing a uniform gradient distribution (Figure 4e). In practice, we used the fluorescent molecule Rhodamine B as a model drug (Figure S4a,b, Supporting Information). The results demonstrated a uniform gradient in fluorescence intensity across the terminal branch channels, consistent with the simulated concentration gradients. This confirmed that the microfluidic chip was capable of generating the concentration gradients necessary for drug screening.

After integrating the microcapsules into the culture chambers, the chip system was bonded to ensure a relatively sealed environment for cell cultivation and subsequent drug evaluation. We validated the chip's capacity to support the viability of cell microcapsules. Throughout the cell culture process, the culture medium was introduced into the chip through its inlet, traversed the branching channels to reach the cell culture chambers, and was ultimately expelled through the outlet of chip. Quantitatively, we recorded the viability of cells on‐chip and compared it to those cultured in a dish, and found that cell growth within the chip could be maintained as expected (Figure 4f). More intriguingly, the medium within the chip remained in a dynamic state of flux, which facilitated the delivery of nutrients and oxygen to the cells within the microcapsules and in removing metabolic waste.

Furthermore, to substantiate the in vitro simulation efficacy of cell spheroids cultured in the microcapsules on‐chip, validation was conducted at the histological level. After a 5‐day cultivation, we subjected the microcapsules to Hematoxylin and Eosin (H&E) staining on the microcapsules to verify the tissue‐level characteristics of the cell spheroids (Figure 4g), and examined the overall and surface features of the cell microcapsules using SEM (Figure 4h). We observed spheroid‐like cell aggregates, with distinct cellular morphologies on the surface. Subsequently, we assessed the expression of Vimentin, Cluster of Differentiation 10 (CD10), and Cluster of Differentiation 44 (CD44) in the hESCs spheroids using immunofluorescence staining, thereby confirming their pathological features (Figure 4i). Vimentin, a component of the cytoskeleton, plays a significant role in the process of epithelial‐mesenchymal transition.^[^ ^24^, ^25^ ^]^ CD10 serves as a pathological diagnostic marker for EMs.^[^ ^26^ ^]^ In the lesion sites of EMs patients, elevated expression levels of CD44 have been documented.^[^ ^27^, ^28^ ^]^ The immunofluorescence staining of the cell spheroids revealed the expression of these proteins. Furthermore, by conducting immunohistochemical staining on both the hESCs spheroids and tissue sections from the corresponding patients, we observed a high level of consistency (Figure S5, Supporting Information).

**Figure 5:**
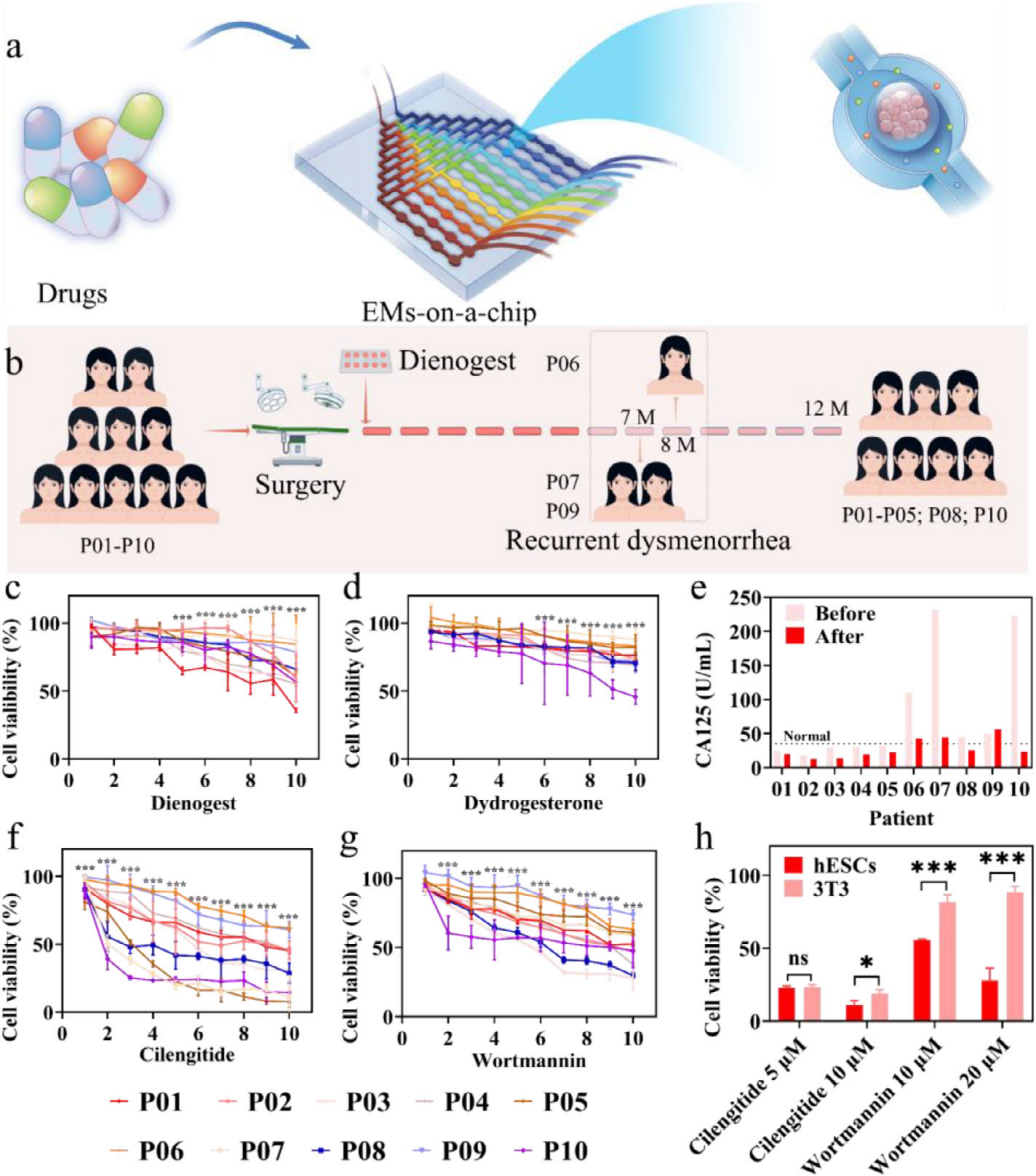
The EMs‐on‐a‐chip platform for drug screening. a) Schematic representation of the drug responses of the microcapsules in the microfluidic chip. b) Schematic of the 12‐month postoperative follow‐up of ten EMs patients (by figdraw.com). c, d) Cell viability on‐chip for 10 patients following a 24‐h (c) dienogest and (d) dydrogesterone treatment. e) Serum CA125 levels from 10 patients before surgery and at the six‐month postoperative. (f, g) Cell viability on‐chip for 10 patients following a 24‐h (f) cilengitide and (g) wortmannin treatment. h) Effect of cilengitide and wortmannin for 24 h on the viability of hESCs and 3T3 cells. (*n* = 30 from ten chips for each group in c, d, f, g. *n* = 6 for each group in h).

Afterward, we evaluated the efficacy of the EMs‐on‐a‐chip for in vitro drug assessment. Initially, we extracted primary ESCs from ten patients with EMs and loaded them into microcapsules, designated as P01 to P10. The clinical data of these ten patients were presented in Table S1 (Supporting Information). These microcapsules were then cultured in a medium for five days to achieve relatively uniform size consistency. Ultimately, the microcapsules were integrated into the chip's cell culture chambers for drug response assays (**Figure** **5**a), with three microcapsules placed in three chambers of each channel, allowing a single chip to accommodate a total of 30 microcapsules for drug reaction evaluation.

Taking the classical oral therapeutic agent dienogest and dydrogesterone as examples, we validated the drug assessment capability of the microfluidic platform. Specifically, we introduced dienogest (1 µM) or dydrogesterone (3 µM) into the chip via one inlet, while simultaneously injecting the culture medium through another inlet. This setup exposed the cells in the microcapsules to circulating drug solutions of varying concentrations. After a 24‐h drug incubation, we retrieved the microcapsules from the cell culture chambers to evaluate cell proliferation. The results indicated that dienogest showed more pronounced inhibitory effect on the proliferation of patient‐derived spheroids from P01, P02, P03, P04, P05, P08, and P10, whereas the inhibition was not apparent in P06, P07, and P09 (Figure 5c). In contrast, dydrogesterone significantly suppressed proliferation only in P10, with the other nine groups showing no significant response to the drug (Figure 5d).

To validate the high‐fidelity simulation of the in vivo environment by the microfluidic chip platform, we correlated the clinical medication profiles of patients with the aforementioned drug responses on‐chip. All patients maintained oral dienogest treatment post‐surgery, and during long‐term follow‐up, normalization of serum Cancer Antigen 125 (CA125) levels was observed in all patients one month post‐operation. However, during the 6‐month follow‐up, patients P06, P07, and P09 exhibited a re‐elevation of serum CA125 beyond the normal range (Figure 5e), suggesting a potential disease recurrence. Patients P07 and P09 experienced the recurrence of dysmenorrhea at seven months post‐surgery, while patient P06 had a recurrence at eight months post‐surgery. The combination of clinical symptoms and laboratory findings for these three patients indicated a relapse of the disease. In contrast, the remaining patients exhibited no evidence of recurrence throughout the 12‐month follow‐up duration (Figure 5b). These results aligned with the on‐chip demonstrations.

The chip‐based drug inhibition assay showed a dose‐dependent trend, with increased dienogest concentrations leading to significant suppression of cell proliferation (Figure 5c) in P01, P02, P03, P04, P05, P08, P10. However, even the maximum drug concentration used on‐chip exerted a weak inhibitory effect on the cell spheroids derived from P06, P07, P09, suggesting that it might lead to temporary alleviation of disease, but not enough to control disease progression, leading to recurrence (Figure 5b). This outcome was consistent with clinical results. These findings suggested that integrating microfluidic chip with hESCs‐encapsulated microcapsules and combining the effect of concentration gradients could effectively simulate the in vivo dynamics of EMs. Consequently, the microcapsule‐integrated microfluidic chip platform system holds significant potential for personalized drug screening in EMs.

Subsequently, we evaluated the capability of the microfluidic chip for the assessment of potential drug for EMs by introducing cilengitide and wortmannin to the platform. Cilengitide, an integrin antagonist known for its pro‐apoptotic and anti‐angiogenic properties, has been investigated for the treatment of glioblastoma and various cancers.^[^ ^29^, ^30^ ^]^ However, its application in EMs has rarely been reported. Wortmannin, a steroidal metabolite derived from fungal culture filtrates, possesses anti‐inflammatory properties and selectively targets phosphoinositol‐3 kinase.^[^ ^31^ ^]^ Studies have demonstrated that wortmannin significantly reduces the viability of EMs cells.^[^ ^32^ ^]^ The drug responses on the chip demonstrated variability in sensitivity to cilengitide and wortmannin among cell spheroids derived from different patients, with several groups exhibiting more pronounced response to the drugs (Figure 5f,g). Moreover, we compared the responses of hESCs and mouse embryonic fibroblast cells (NIH 3T3), which represent cells of normal tissue, to cilengitide and wortmannin. Notably, an intriguing phenomenon was observed: at relatively low drug concentrations, cilengitide exhibited equivalent inhibitory effects on 3T3 and hESCs; at relatively high concentration, the inhibitory effect on both cell types was further enhanced. Wortmannin showed a dose‐dependent inhibitory effect on hESCs but had no obvious effect on 3T3 cell (Figure 5h). Consequently, we hypothesized that the EMs‐on‐a‐chip platform can help evaluate potential drugs with a targeted therapeutic effect specific to EMs.

**Figure 6:**
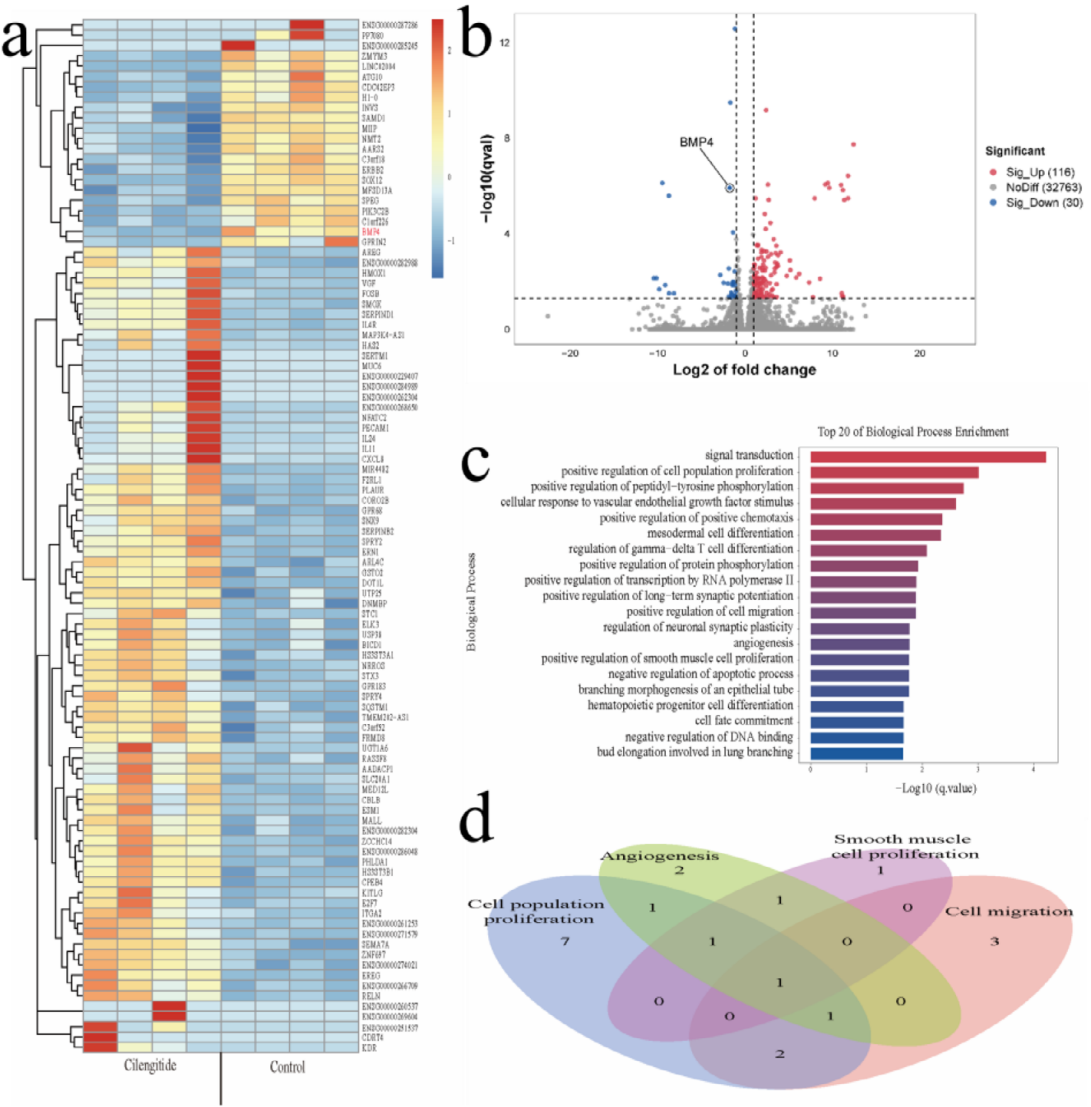
Differential transcriptomic profiles of hESCs after drug treatment. a) Following the administration of cilengitide, heatmap depicting the Differentially Expressed Genes of top 100 (DEGs): each small square represents an individual gene, with the color intensity reflecting the gene's expression level. b) Volcano plot of DEGs: Red dots denote genes with increased expression; blue dots highlight those with decreased expression, while gray dots reflect genes showing no significant change in expression levels. c) Top 20 GO (Biological Process) Entries. The vertical axis represents each GO term, sorted by q‐value. The x‐axis denotes the proportion of genes linked to each term. The column color corresponds to the q‐value, with a deeper red hue indicates greater reliability. The column length corresponds to the number of genes involved, with longer columns indicating more genes implicated. d) Venn diagram of overlapping gene signatures in cell proliferation, migration, and angiogenesis. (*n* = 4).

Furthermore, we demonstrated the integration of the EMs‐on‐a‐chip platform with omics techniques to further investigate drug efficacy. We conducted transcriptome sequencing on hESCs spheroids from four groups before and after drug treatment on the chip. The results suggested that cilengitide may inhibit the proliferation of hESCs through pathways associated with cell proliferation, migration, and angiogenesis, with the bone morphogenetic protein 4 (BMP4) mRNA involved in regulating these pathways (**Figure** **6**a–d; Figure S7a–c, Supporting Information). BMP4, a component of the Transforming Growth Factor‐beta (TGF‐β) superfamily, is regulated by β‐catenin and has been shown to modulate cell apoptosis, with potential implications for the pathogenesis of EMs.^[^ ^33^, ^34^ ^]^ Our research findings indicated that following cilengitide treatment on‐chip, the expression of BMP4 in hESCs was downregulated, and the proliferation of the majority of cell spheroids was inhibited. This aligned with the conclusions from previous in vivo studies,^[^ ^33^, ^34^ ^]^ thereby confirming the reliability of the drug assessment results obtained from the chip platform.

In contrast, Wortmannin primarily inhibited the proliferation of hESCs through pathways associated with apoptosis, with the regulation of both BCL‐2 binding component 3 (BBC3), and BCL‐2 like protein 11 (BCL2L11, also known as BIM) mRNA implicated in these pathways (Figures S6a–d and S8a–c, Supporting Information). BBC3 and BIM are members of the BCL‐2 family and serve as crucial regulators of apoptosis. Studies have demonstrated that the expression of BBC3^[^ ^35^, ^36^ ^]^ and BIM^[^ ^35^, ^36^ ^]^ mRNA is reduced in hESCs and EMs tissues. Here, we found that, following wortmannin treatment on the chip, there was an upregulation of BBC3 and BIM mRNA in hESCs, and the proliferation of cell spheroids was inhibited. This further confirmed the capability of the chip platform to efficiently explore the therapeutic potential of drugs for EMs and analyze their mechanisms of action.

## 3 Conclusion

In conclusion, this study developed an innovative microfluidic EMs‐on‐a‐chip platform for drug evaluation. First, we constructed a coaxial microfluidic electrospray device. Through the adjustment of multiple parameters, we fabricated microcapsules featuring a CMC core and crosslinked ALG shell, which were uniform and biocompatible. These microcapsules were used to load primary hESCs for 3D culture, ultimately forming cell spheroids. These cell spheroids were then integrated into a multi‐channel microfluidic chip platform with a concentration gradient generator for drug screening. The chip platform facilitated nutrient transport and drug diffusion, allowing for the in vitro culture model to simulate the in vivo conditions at the histological level. The differential drug responses observed among patients may be attributed to genetic polymorphisms and epigenetic variations between individuals. In line with clinical responses from corresponding patients, on‐chip experimental results revealed personally varying drug sensitivities. This highlighted the platform's efficient drug screening capability. Taking cilengitide and wortmannin as examples, we validated the platform's potential for drug discovery after integrating with advanced technologies like omics. Furthermore, the unique branched structure of the microfluidic chip enables generation of concentration gradients. By modifying the drug composition, concentration, and exposure time, a variety of combination drug treatment protocols can be developed for drug screening, thereby enhancing the therapeutic efficiency of endometriosis.

In summary, our microfluidic chip platform efficiently mimics the in vivo biological environment of EMs, showing great promise in addressing challenges in EMs drug evaluation, screening, and disease therapy. The microfluidic chip platform establishes personalized drug sensitivity profiles, facilitating data‐driven optimization of therapeutic regimens. Notably, for patients with drug resistance and high recurrence rates, the microfluidic chip platform can be utilized to explore effective therapeutic strategies, providing informative guidance and avoiding unnecessary drug exposure. Future work will involve the incorporation of more components of the in vivo microenvironment, such as vascular structures, immune cell interactions, and neuroendocrine factors, into the microfluidic chip platform to more accurately simulate the in vivo conditions. Additionally, efforts will be made to expand the sample size to conduct a large‐scale validation of the platform's drug screening capabilities.

## 4 Experimental Section

### Materials

ALG, CMC, CaCl_2_, dienogest, and dydrogesterone powders were procured from Macklin. Fluorescent polystyrene nanoparticles were sourced from Thermo Fisher. Cilengitide and wortmannin powders were acquired from MCE. Capillary glass tubes were acquired from Nantong Hairui Laboratory Equipment Co., Ltd. The CCK‐8 assay kit and Live/Dead cell staining kit were obtained from Beyotime. Type IV Collagenase was acquired from Gibco. Cell culture plates were obtained from Nest Life Science Technology Co. Ltd. Both primary (Vimentin, CD10, and CD44) and secondary antibodies (Anti‐Rabbit, Anti‐Mouse) were procured from Proteintech.

### Fabrication of Hydrogel Microcapsules

A coaxial microfluidic setup, consisting of an inner and outer capillary glass tube, was constructed to produce microcapsules via electrospray. The inner tube had a diameter of ≈100 µm, and the outer tube measured ≈300 µm in diameter. The tubes were affixed to a glass slide using epoxy resin, ensuring their coaxial positioning in both the horizontal and vertical axes. The inner phase consisted of a 1 wt% CMC solution, while the outer phase was composed of a 1.5 wt% ALG solution. By applying an electric field between the device and the collection medium, the liquids were transformed into microdroplets with CMC at the core and ALG forming the shell. The dimensions of the resulting microcapsules were meticulously regulated by fine‐tuning variables including the outer and inner phase flow rates, the intensity of the applied electric field, and the position of the collection liquid.

### Human EMs Specimen

Specimens for the study of EMs were sourced from ten individuals afflicted with the condition, all of whom were treated surgically at the Second Affiliated Hospital of Wenzhou Medical University. The specimens underwent histological assessment, which confirmed the diagnosis of EMs, and the cyst wall tissues collected from these ovarian lesions were histologically verified as endometrial ectopic implants. The endometriosis tissue specimens obtained from surgery were placed into sterile centrifuge tubes containing PBS and immediately transported to the laboratory on ice for cell extraction. Informed consent was obtained from each participant. The Ethics Committee of the Second Affiliated Hospital of Wenzhou Medical University approved the study (2025‐K‐13‐01).

### Extraction of hESCs

The EMs tissue underwent triple rinsing with cold phosphate buffered saline (PBS), followed by mincing into fragments and incubation with twice the volume of Type IV Collagenase with a water bath shaker at 37 °C for 60 min. After enzymatic digestion, the reaction was terminated by the addition of serum. Following centrifugation of the cell suspension at 1800 rpm, 4 °C for 5 min. After carefully removing the supernatant, the cells were resuspended in fresh culture medium and then plated into culture dishes to facilitate further growth.

### Fabrication of Microcapsules Containing hESCs

Cultured hESCs were treated with trypsin to obtain a cell suspension, which was then combined with a 1 wt% CMC solution and introduced into the inner capillary of the microfluidic coaxial. Concurrently, a 1.5 wt% ALG solution was delivered into the outer capillary. To ensure uniformity among cell microcapsules derived from different patients, the cell density within the CMC solution, the flow rates of the inner and outer phases, the height of the collection liquid, and the applied voltage were all maintained at consistent levels during the microcapsule synthesis process. The resultant cell‐laden microcapsules were subsequently transferred to the culture medium and placed in an incubator for culture.

### Cultivation of hESCs‐Laden Microcapsules

The complete culture medium was established by incorporating 10% fetal bovine serum and 2% penicillin‐streptomycin solution into DMEM medium. The hESCs‐laden microcapsules were then cultured in this complete DMEM medium for 5 days, with medium changes performed every 48 h.

### Cell Viability Assay

Cell viability within the hydrogel microcapsules was evaluated utilizing a live/dead cell staining assay. After retrieving the cultured cell‐laden microcapsules from the medium and rinsing them with PBS, the live/dead staining solution was introduced and allowed for a 30‐min incubation period in a dark, 37 °C setting. Live cells could be visualized in green, and dead cells in red. A quantitative assessment of cell viability inside the microcapsules was conducted employing the CCK‐8 assay kit. Each well of a 96‐well plate received 10 cell microcapsules, after which 100 µL of CCK‐8 reagent was added. Incubation occurred at 37 °C for a duration of 2 h. Subsequently, the absorbance was determined with the aid of a plate reader at 450 nm.

### Immunohistochemistry of Human EMs Tissues

The harvested tissue was fixed in formalin, followed by dehydration and embedding of the specimens in paraffin. After sectioning, the slides were deparaffinized, and immunohistochemical staining was conducted using antibodies against Vimentin (1:4000), CD10 (1:500), and CD44 (1:200).

### Immunofluorescence of EMs Cell‐Laden Microcapsules

hESCs‐laden microcapsules were fixed with paraformaldehyde for 2 h, followed by permeabilization with 0.1% Triton X‐100. To prevent nonspecific antibody binding, the samples were incubated with a 5% bovine serum albumin solution. Following this, overnight incubation of the microcapsules at 4 °C was performed with primary antibodies specific to Vimentin (1:500), CD10 (1:200), and CD44 (1:200). Following an overnight incubation, the microcapsules were treated with fluorescently labeled secondary antibodies for 1 h and then incubated with 4′,6‐diamidino‐2‐phenylindole (DAPI) for 10 min. The antibodies used were fluorescently labeled: anti‐mouse IgG (Proteintech, DyLight‐488, 1:500) and anti‐rabbit IgG (Proteintech, CL488, 1:500).

### Characterization

The microcapsules underwent supercritical processing, platinum sputter coating, and were subsequently examined using a scanning electron microscope SEM (SU8010, HITACHI). hESCs‐laden microcapsules were fixed using 4% paraformaldehyde for a duration of 2 h, followed by a series of graded ethanol dehydrations and supercritical drying, and subsequently examined using the SEM. Confocal laser scanning microscopy (FV3000, Olympus) was utilized to capture fluorescence images of the cells encapsulated within the microcapsules.

### Design of Microfluidic Chip with Concentration Gradient

The polydimethylsiloxane chip was crafted employing conventional soft lithography methods. It included dual liquid inlets, a concentration gradient generator, and a drug reaction chamber. The concentration gradient generation module utilized a dendritic branching pattern. The drug reaction module comprised 10 effluent channels, with each channel featuring 3 cell culture chambers. The microfluidic chip was fabricated with standardized geometric configurations.

### On‐Chip Drug Assessment

hESCs‐laden microcapsules, after a 5‐day culture period, were relocated into the cell culture chambers of the microfluidic chip, with a single microcapsule in each chamber. Following this, the chip was sealed and the drug was introduced into it to initiate the reaction. Post a 24‐h incubation, the cell‐laden microcapsules were retrieved, and the inhibitory effects of the drug were assessed employing the CCK‐8 assay kit in accordance with the manufacturer's guidelines.

### Transcriptome Sequencing

Endometriosis stromal cells were processed to extract total RNA via Trizol. The purified RNA fragments were reverse‐transcribed into cDNA, and double‐stranded DNA was synthesized thereafter. Subsequently, RT‐PCR amplification was conducted to assemble a library featuring an average fragment size of 300 bp ± 50 bp. Subsequently, the library was then processed for paired‐end sequencing on the platform of Illumina Novaseq 6000, adhering to the established protocols.

### Statistical Analyses

The data presented were normalized relative to the control group. Data are presented as means ± SD. Statistical analyses were conducted using the Student's *t*‐test. The sample size (*n*) is specified in the figure legends. “NS” denotes no significant difference. Statistical significance levels are denoted as ^*^ *p* < 0.05, ^**^ *p* < 0.01, or ^***^ *p* < 0.001, with further details provided in the figure legends. Bioinformatics analyses were conducted, and the Wilcoxon test was utilized for inter‐group comparisons.

## Conflict of Interest

The authors declare no conflict of interest.

## Supporting information



Supporting Information

## Data Availability

The data that support the findings of this study are available from the corresponding author upon reasonable request.
