# Supplementary material for: Drug Screening of Primary Human Endometriotic Cells Based on Micro‐Encapsulating Microfluidic Chip
Source: Adv Sci (Weinh). 2025 Apr 28;12(20):2504647. doi: 10.1002/advs.202504647 (PMC12120730; doi:10.1002/advs.202504647)
Supplement: Supplementary file 1 — Supporting Information [file ADVS-12-2504647-s001.docx]

Supporting Information

Drug screening of primary human endometriotic cells based on micro-encapsulating microfluidic chip

Qiong Chen, Jing Wang, Wenzhao Li, Luoran Shang*, Dexuan Wang*, Ping Duan*

**Table S1.** Preoperative profiles of ten patients diagnosed with EMs.

| Patient | Stage | CA125 （U mL^-1^）（0~35 U mL^-1^） |
| --- | --- | --- |
| P01 | IV | 24.4 |
| P02 | III | 18.2 |
| P03 | IV | 29.1 |
| P04 | IV | 30.3 |
| P05 | IV | 31.8 |
| P06 | IV | 110 |
| P07 | IV | 232 |
| P08 | IV | 44.8 |
| P09 | IV | 39.4 |
| P10 | IV | 223 |


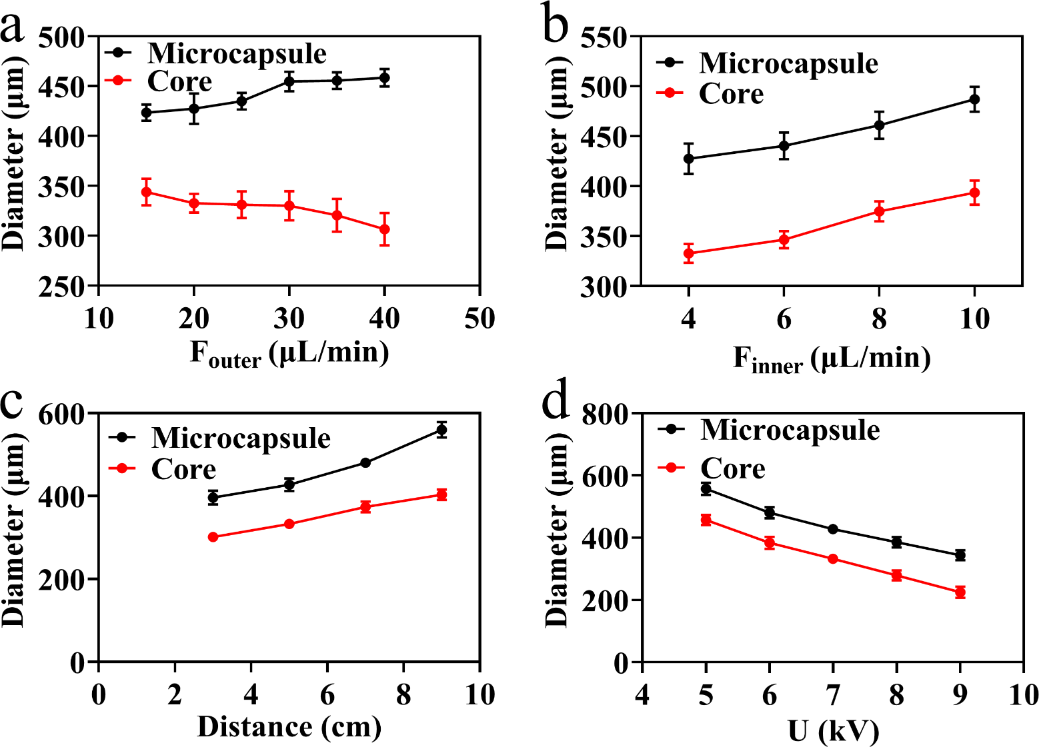


**Figure S1**. The variation in microcapsule dimensions influenced by the external flow rate and internal flow rate, the position of the receiving liquid, and the applied voltage. a) An increase external flow rate results in larger microcapsule size and smaller core diameter. b) As the internal flow rate is elevated, the dimensions of the microcapsules and their core diameters correspondingly increased. c) Increasing the collection distance causes a corresponding increase in microcapsule dimensions and core diameters. (d) Increasing the voltage causes a corresponding decrease in microcapsule dimensions and core diameters. (a-d, n=100 for each group).


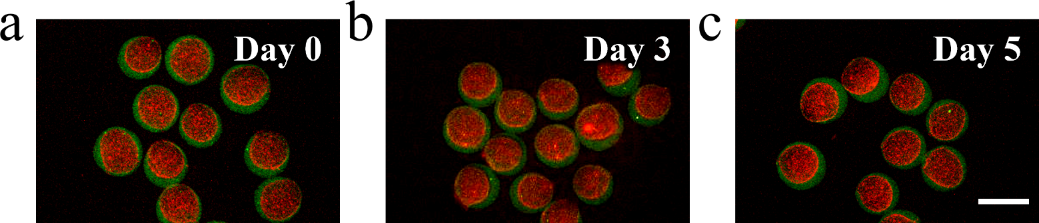


**Figure S2**. Microscopic images of microcapsules in the cell culture medium on day (a) 0, (b) 3, and (c) 5. Scale bar: 500 μm.


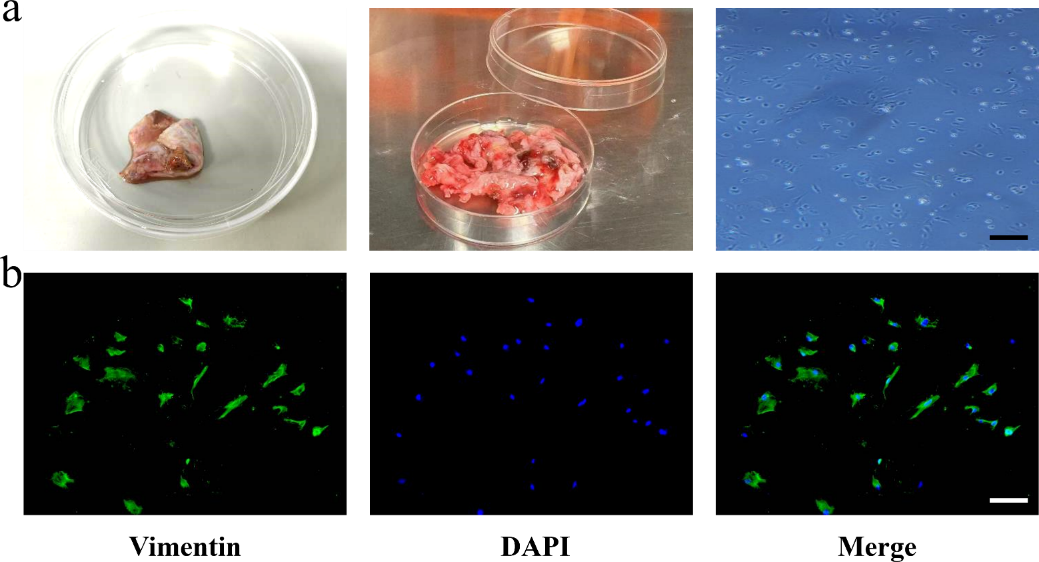


**Figure S3**. Extraction and identification of primary human hESCs. a) Isolation and extraction of hESCs. b) Identification of stromal cells in EMs. The scale bars are 50 μm.


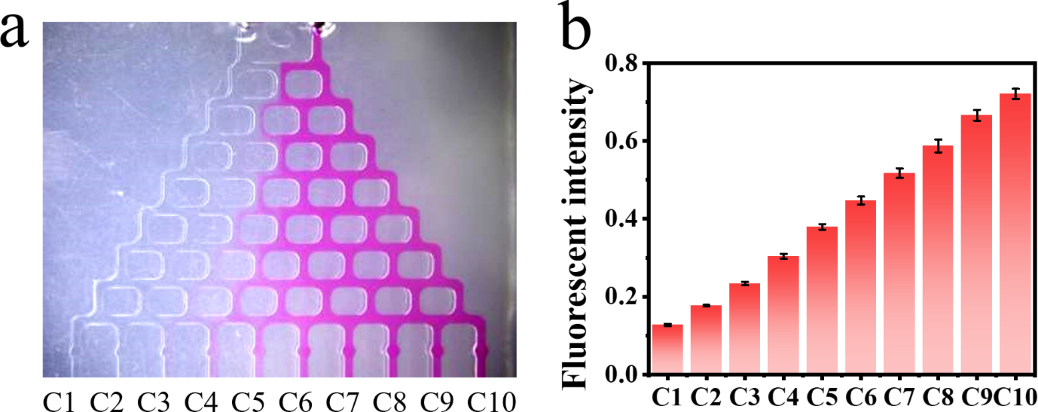


**Figure S4.** Concentration gradient distribution within the microfluidic chip. a) Optical microscopic image of the distribution of Rhodamine B within the microfluidic chip channels. A 100 μM solution of Rhodamine B was introduced through the left inlet at a 0.2 μL min^-1^ flow rate, while a PBS solution was simultaneously introduced through the right inlet at an identical flow rate. b) The fluorescent intensity of Rhodamine B quantified across the terminal branches of the microfluidic channel, from C1 to C10. (n=6 for each group).


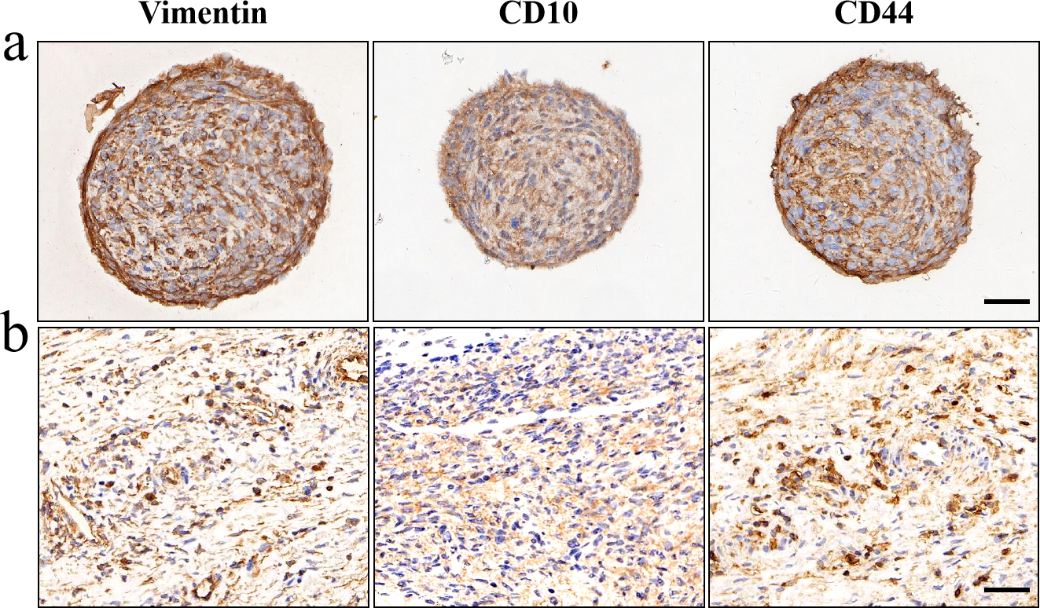


**Figure S5**. Immunohistochemical images of (a) hESCs spheroids and (b) Ovarian endometriotic cyst tissues for Vimentin, C10, and CD44. The scale bar is 50 μm.


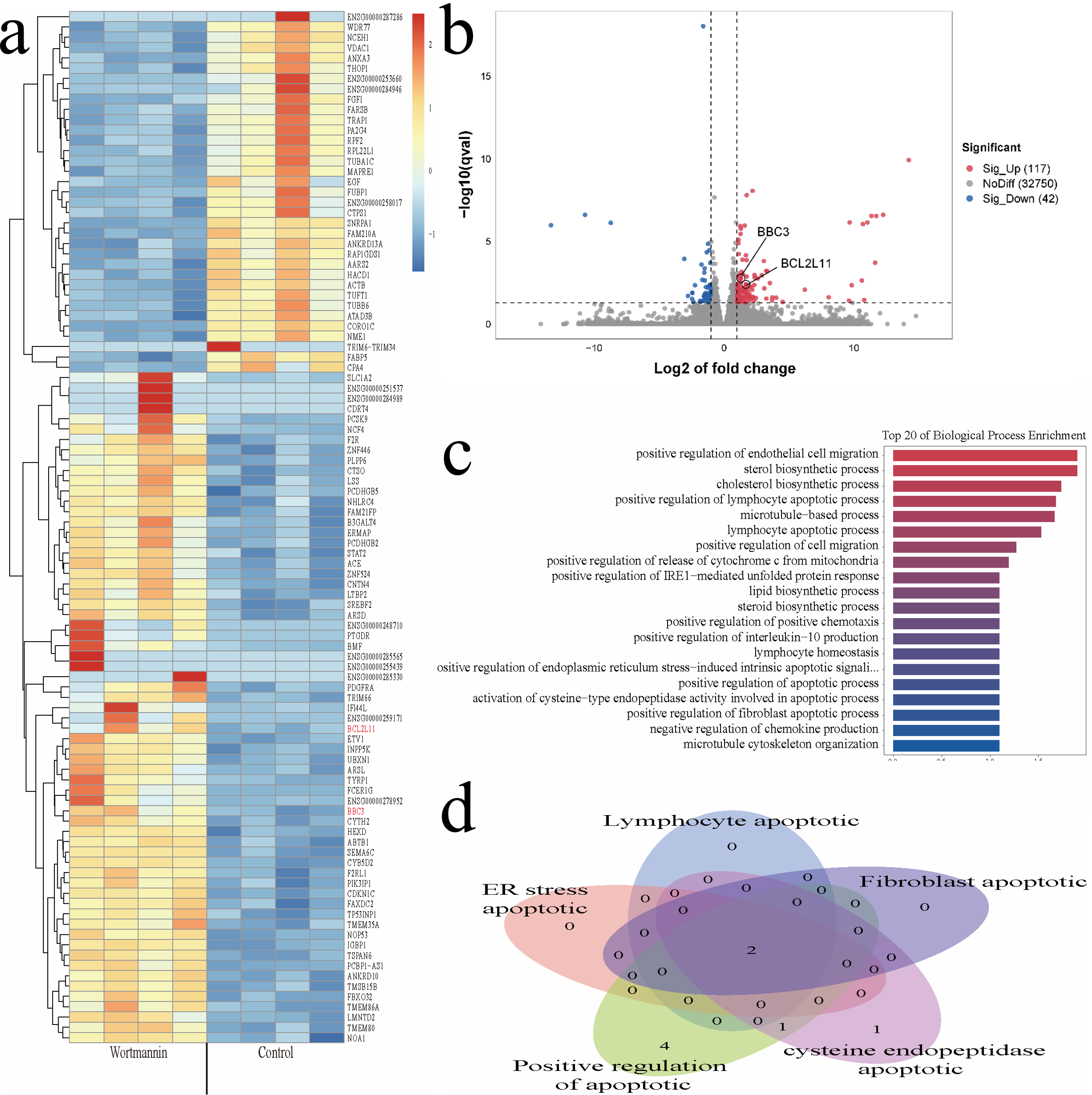


**Figure S6.** a) Following the administration of wortmannin, heatmap of the top 100 DEGs. b) Volcano plot of DEGs. c) Top 20 GO (Biological Process) Entries. d) Venn diagram depicting the signature genes across 5 key apoptosis-associated pathways. (n=4)

**
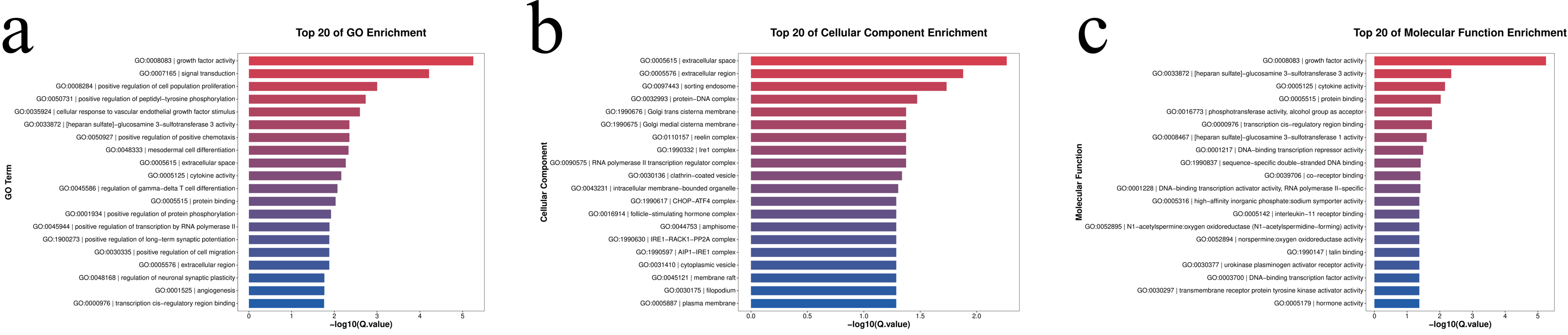
**

**Figure S7.** a-c) Differential transcriptomic profiles of hESCs pre- and post-cilengitide treatment. GO Term entries of top 20 (a). GO Cellular Component entries of top 20 (b). GO Molecular Function entries of top 20 (c). The vertical axis represents each GO term, sorted by q-value. The x-axis denotes the proportion of genes linked to each term. The column color corresponds to the q-value, with a deeper red signifying higher reliability. The column length corresponds to the number of genes involved, with longer columns indicating more genes implicated.

**
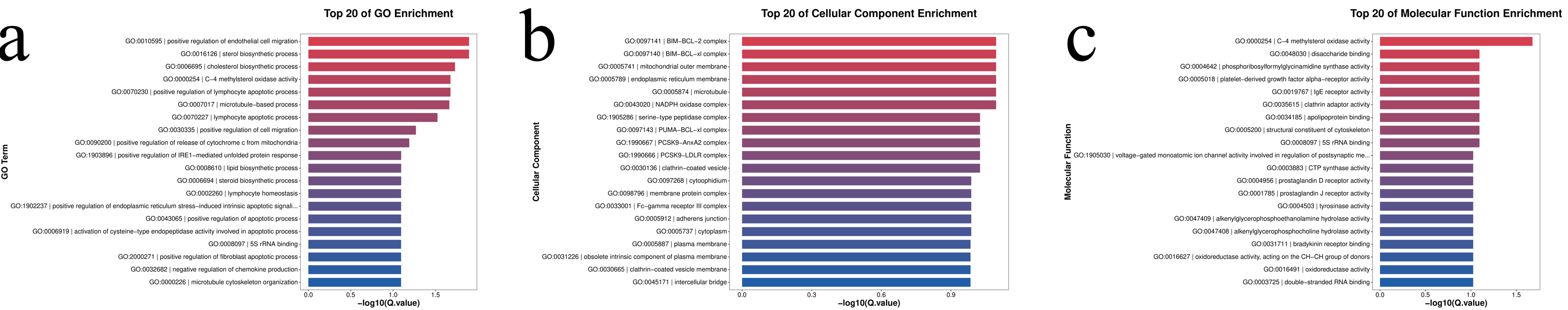
**

**Figure S8.** a-c) Differential transcriptomic profiles of hESCs pre- and post-Wortmannin treatment. GO Term entries of top 20 (a). GO Cellular Component entries of top 20 (b). GO Molecular Function entries of top 20 (c). The vertical axis represents each GO term, sorted by q-value. The x-axis denotes the proportion of genes linked to each term. The column color corresponds to the q-value, with a deeper red signifying higher reliability. The column length corresponds to the number of genes involved, with longer columns indicating more genes implicated.
